# General regulatory factors exert differential effects on nucleosome sliding activity of the ISW1a complex

**DOI:** 10.1186/s40659-024-00500-6

**Published:** 2024-05-04

**Authors:** Andrea Oyarzún-Cisterna, Cristián Gidi, Fernanda Raiqueo, Roberto Amigo, Camila Rivas, Marcela Torrejón, José L. Gutiérrez

**Affiliations:** Departamento de Bioquímica y Biología Molecular, Facultad de Ciencias Biológicas, Universidad de Concepción, 4070043 Concepción, Chile

**Keywords:** Chromatin remodeling, ISW1a, Nucleosome sliding, Rap1, Nucleosome remodeling

## Abstract

**Background:**

Chromatin dynamics is deeply involved in processes that require access to DNA, such as transcriptional regulation. Among the factors involved in chromatin dynamics at gene regulatory regions are general regulatory factors (GRFs). These factors contribute to establishment and maintenance of nucleosome-depleted regions (NDRs). These regions are populated by nucleosomes through histone deposition and nucleosome sliding, the latter catalyzed by a number of ATP-dependent chromatin remodeling complexes, including ISW1a. It has been observed that GRFs can act as barriers against nucleosome sliding towards NDRs. However, the relative ability of the different GRFs to hinder sliding activity is currently unknown.

**Results:**

Considering this, we performed a comparative analysis for the main GRFs, with focus in their ability to modulate nucleosome sliding mediated by ISW1a. Among the GRFs tested in nucleosome remodeling assays, Rap1 was the only factor displaying the ability to hinder the activity of ISW1a. This effect requires location of the Rap1 cognate sequence on linker that becomes entry DNA in the nucleosome remodeling process. In addition, Rap1 was able to hinder nucleosome assembly in octamer transfer assays. Concurrently, Rap1 displayed the highest affinity for and longest dwell time from its target sequence, compared to the other GRFs tested. Consistently, through bioinformatics analyses of publicly available genome-wide data, we found that nucleosome occupancy and histone deposition in vivo are inversely correlated with the affinity of Rap1 for its target sequences in the genome.

**Conclusions:**

Our findings point to DNA binding affinity, residence time and location at particular translational positions relative to the nucleosome core as the key features of GRFs underlying their roles played in nucleosome sliding and assembly.

**Supplementary Information:**

The online version contains supplementary material available at 10.1186/s40659-024-00500-6.

## Background

Chromatin dynamics is deeply involved in transcriptional regulation, as well as in other processes that require access to DNA. Among the factors playing a role within chromatin dynamics are chromatin remodeling complexes (CRCs), DNA sequences and transcription factors [1, 2]. These factors act concertedly to establish and maintain defined nucleosome positioning patterns. In this regard, genome-wide analyses performed in different species have found a positioning pattern surrounding transcription start sites (TSSs), shared by a large number of genes, consisting in a nucleosome-free region (NFR) or nucleosome-depleted region (NDR) flanked upstream and downstream by well positioned nucleosomes named − 1 and + 1 nucleosomes, respectively. The pattern is completed by a number of nucleosomes spaced by equal lengths of linker DNA, downstream the + 1 nucleosome [1, 3, 4]. In the yeast *Saccharomyces cerevisiae*, this regular spacing is catalyzed by CRCs such as Ino80, ISW1a/b, ISW2 and Chd1 [5–7]. It has been observed that the position of the + 1 nucleosome, which stands as the border from where the regular spacing is established, relies on the presence of a barrier located in the NDR. This barrier consists in DNA sequences that directly influence the action of particular CRCs or serve as binding sites for defined transcription factors [1, 7–10], termed General Regulatory Factors (GRFs) or Nucleosome-Displacing Factors (NDFs) in yeast and Pioneer Factors (PFs) in higher eukaryotes [11].

In *S. cerevisiae*, Reb1, Abf1, Cbf1 and Rap1 are among the main proteins characterized as GRFs [12]. Their property of acting as barriers contributes to the maintenance of NDRs, and might contribute to NDR formation as well [4, 13, 14]. It is currently conceived that a given GRF bound to its cognate sequence in an NDR acts as an obstacle for the sliding activity of CRCs that tend to mobilize nucleosomes into this region [4, 13] and might interfere with histone deposition [12]. Both properties would rely on high affinity of the GRF to its target sequence and/or low dissociation rates [12, 15–17]. In this context, it has been determined that Reb1 and Cbf1 bias the activity of Chd1 in such a way that nucleosomes are slid away from their binding sites [17]. Similarly, the DNA binding domain (DBD) of the transcription factor Gal4 has the ability to hinder the sliding activity of the ISW1a complex [15]. Besides their modulatory effect on Chd1, Reb1 and Cbf1 interfere with histone deposition in chaperone-mediated nucleosome assembly assays [17].

Among CRCs whose activity results in nucleosomes populating NDRs is the ISW1a complex [18]. Although several in vivo genome-wide studies had demonstrated the ability of the aforementioned GRFs in establishment and maintenance of NDRs, there are no analyses directly assessing their effect on ISW1a nucleosome sliding activity. In addition, comparative analyses of the biochemical properties of these GRFs involved in their function as a barrier for sliding activity have not been performed. Considering this scenario, in this work we comparatively analyzed the influence Reb1, Abf1, Cbf1 and Rap1 on ISW1a sliding activity. Additionally, we compared the affinity and dissociation rates of these GRFs. Among the GRFs studied, only Rap1 displayed the property of hindering ISW1a-mediated nucleosome sliding. Consistently, this GRF displayed the highest affinity for its cognate sequence and the lowest dissociation rate. In addition, Rap1 showed the ability to hinder nucleosome assembly in octamer transfer assays. These properties were strictly dependent on binding of Rap1 to its target sequence in defined positions relative to the nucleosome core. Our results define DNA binding affinity, residence time and location at particular translational positions as the key properties of GRFs involved in the roles that these factors play in nucleosome sliding and assembly.

## Results

### Rap1 hinders nucleosome sliding mediated by ISW1a

**Fig. 1:**
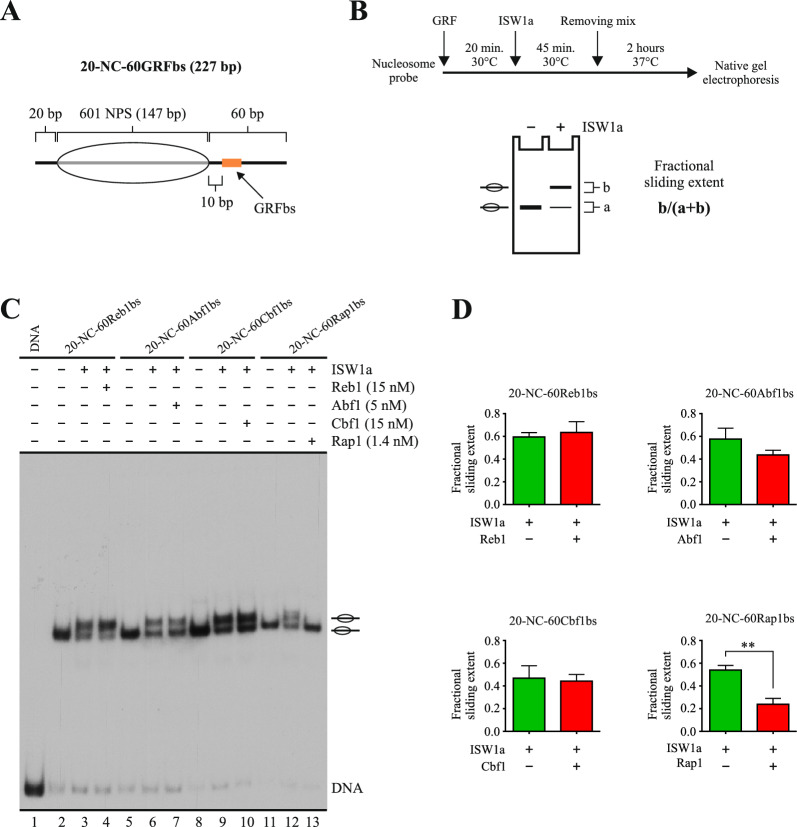
Among the GRFs tested, only Rap1 has the ability to hinder ISW1a sliding activity. **A** Schematic representation of the nucleosome probes used in the assays. 601 NPS = nucleosome positioning region of the 601 sequence (gray bar; 147 bp). The oval represents the translational position adopted by the nucleosome core upon reconstitution, which covers the 601 region. The term “NC” in probe names stands for “nucleosome core”. Probe names indicate length of linker DNA upstream (left) and downstream (right) of the NC and presence of a defined GRF binding site (GRFbs). **B** Upper panel: outline of the steps involved in the nucleosome remodeling assay. Lower panel: schematic representation depicting the remodeling pattern generated by ISW1a and the method used to quantify its activity. The “fractional sliding extent” corresponds to the ratio of intensity given by bands reflecting remodeled (slid) nucleosome over the intensity given by all bands of the nucleosome probe in the lane. **C** Nucleosome remodeling assay visualized by electrophoresis in a non-denaturing polyacrylamide gel, testing the effect of each GRF on the sliding activity of ISW1a. The probe used in each reaction is depicted at the top of the gel picture, as well as absence or presence of ISW1a (1.5 nM) and a given GRF. The image is representative of three independent assays, performed under the same conditions. Migrations of alternative forms of the nucleosome probe, which correspond to different translational positions of the histone octamer, are indicated schematically at the right of the picture. **D** Quantification of fractional sliding extent and statistical analysis for each probe. Bars in the graphs display the average of three independent assays for each condition analyzed (n = 3). Error bars represent one standard deviation. Asterisks denote statistically significant differences (**p < 0.01), as deducted from a two-tailed unpaired *t*-test

In order to compare the effect that the main GRFs found in *S. cerevisiae* have on the sliding activity of the ISW1a complex, we performed an in vitro sliding assay using four variations of a mononucleosome probe. The probes harbor the 601 nucleosome positioning sequence, in addition to 20 bp of extranucleosomal DNA (from here referred to as linker DNA) upstream and 60 bp downstream this sequence. The sequence of these four probes differs only in a segment located 10 bp downstream the 601 region, where each probe harbors the binding sequence for one of the GRFs tested (Fig. 1A). The GRFs Reb1, Abf1, Cbf1 and Rap1 were obtained as His-tag recombinant proteins using bacterial expression vectors (Additional file 1: Fig. S1). The concentration of each factor in the assay was adjusted in order to obtain 90–95% binding. The steps involved in the assay are depicted in Fig. 1B. As described in this figure, the GRFs were removed from the probes after the step were nucleosome remodeling proceeds, in order to allow visualization of the remodeling pattern on the nucleosomes. The removing was accomplished by adding an excess (100×, relative to GRF concentration) of a non-labeled double-strand oligonucleotide harboring the binding sequence of the corresponding GRF. Short incubation times were sufficient for binding removal of Reb1, Abf1 and Cbf1 (30 min, 30 °C), but the conditions of this step (2 h, 37 °C) were set based on Rap1 behavior, for which a higher temperature and longer incubation period were required to accomplish binding removal (Additional file 1: Fig. S2).

For mononucleosomes harboring linker DNA in both sides of the nucleosome core, it has been described that ISW1a preferentially binds to the longer linker and mobilizes the histone octamer towards this linker. This mobilization continues until the histone octamer reaches a central position in the DNA segment, which is reflected by a slower migration of the nucleosome in native gel electrophoresis [19]. For this sliding assay, this property of the ISW1a complex and the design of the probes—which harbor the GRF binding site in the longer linker—imply that the ISW1a-mediated mobilization of the histone octamer goes in the direction where the GRF is bound. As observed in Fig. 1C, the result of this assay showed that, among the GRFs tested, only Rap1 has the ability to hinder ISW1a sliding activity (Fig. 1C, compare lane 12 to 13), with a reduction to less than half of the activity displayed in the absence of this GRF (Fig. 1D). A slight but reproducible reduction of ISW1a sliding activity was observed in the presence of Abf1, although the difference was not statistically significant (Fig. 1C, D).

### Rap1 displays the highest affinity and lowest dissociation rate among GRFs

**Fig. 2:**
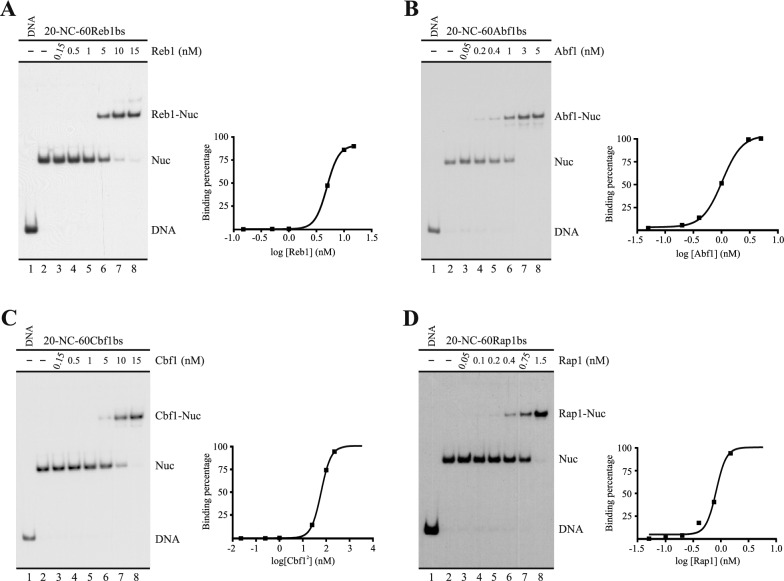
Rap1 displays the highest affinity for its corresponding target sequence. **A**–**D** Apparent Kd determination for Reb1(A), Abf1(B), Cbf1(C) and Rap1(D) factors. The probes and conditions used in the assays are the same used in the remodeling assay shown in Fig. 1, but here the electrophoretic analysis proceeded right after a 30 min incubation with the corresponding GRF. The images correspond to electrophoresis in a non-denaturing polyacrylamide gel and are representative of preliminary assays using similar GRF concentrations under the same conditions. The probe used in each reaction and concentrations of each GRF are depicted at the top of the gel pictures; migrations of free DNA probe (DNA), nucleosome probe (Nuc) and nucleosome probe bound by the corresponding GRF (GRF-Nuc) are indicated at the right of the gel pictures. The graphs at the right of each gel image correspond to densitometric quantification of binding percentages used for Kd determination

**Fig. 3:**
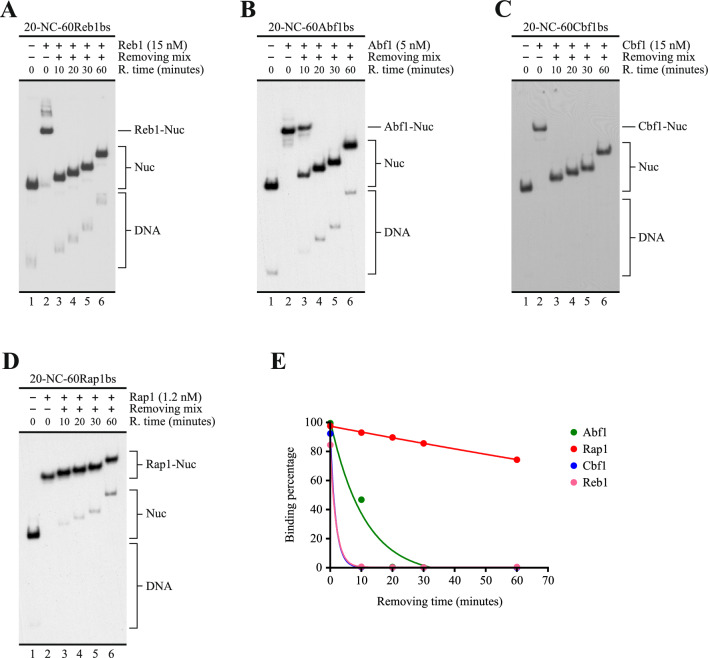
Rap1 displays the longest dwell time at its corresponding target sequence. **A**–**D** Dissociation kinetics analysis for Reb1(A), Abf1(B), Cbf1(C) and Rap1(D) factors. The images correspond to electrophoresis in a non-denaturing polyacrylamide gel analyzing the dissociation extent at different time points after addition of a Removing mix carrying a non-labeled double-stranded oligonucleotide harboring the target sequence for the corresponding GRF (see Methods for details). The probes and chemical conditions used in the assays are the same used in the remodeling and binding assays shown in Figs. 1 and 2. The probe used in each reaction, presence of the corresponding GRF, presence of Removing mix and Removal time points (R. time) are depicted at the top of the gel pictures; migrations of free DNA probe (DNA), nucleosome probe (Nuc) and nucleosome probe bound by the corresponding GRF (GRF-Nuc) are indicated at the right of the gel pictures. **E** Graphical representation of binding percentages observed at each time point. Binding percentages were determined by densitometric quantification of the gel image files

The fact that, among the GRFs tested, only Rap1 was able to hinder ISW1a’s sliding activity, led us to study properties in this factor that could account for this differential effect. In this context, in preliminary work carried out to set the conditions of the aforementioned nucleosome remodeling assay, we noted that Rap1 displayed the highest affinity for its corresponding target sequence, as a very low concentration of this factor was required to reach 90–95% binding, relative to the other GRFs tested. Considering this observation, we aimed to more precisely compare the affinities of these GRFs to their target sequences. To do this, we determined the apparent Kd of these factors by EMSA, using the same probes already used in the sliding assay. Under our assay conditions, we determined Kd values 4.9 nM for Reb1, 1 nM for Abf1, 7.8 nM for Cbf1 and 0.8 nM for Rap1 (Fig. 2). These results confirmed that Rap1 has the higher affinity for its corresponding target sequence, although Abf1 and Rap1 displayed very similar affinities. Considering this, we analyzed whether there is a more pronounced difference between Rap1 and the other GRFs in terms of their dissociation rates. To this respect, previous studies have reported long residence times of Rap1 once bound to its target sequences in the genome [14], which was consistent with the long removal time and elevated temperature that we had to set for our sliding assay to accomplish Rap1 removal (Fig. 1B, Additional file 1: Fig. S2). To test the dissociation rates, we performed time-course analyses, consisting in analyzing the binding percentage remaining after different times of removal incubation. As observed in Fig. 3, for both Reb1 and Cbf1 total removal was accomplished after 10 min (the shortest incubation time, Fig. 3A, C). Total removal of Abf1 binding was accomplished after 20 min of removal incubation (Fig. 3B). Remarkably, Rap1 binding was minimally reduced even under the longest incubation time of the assay (Fig. 3D). Thus, this comparative analysis showed that Rap1 displays a markedly lower dissociation rate, relative to the other GRFs tested.

### Rap1 requires binding on entry DNA to hinder ISW1a activity

**Fig. 4:**
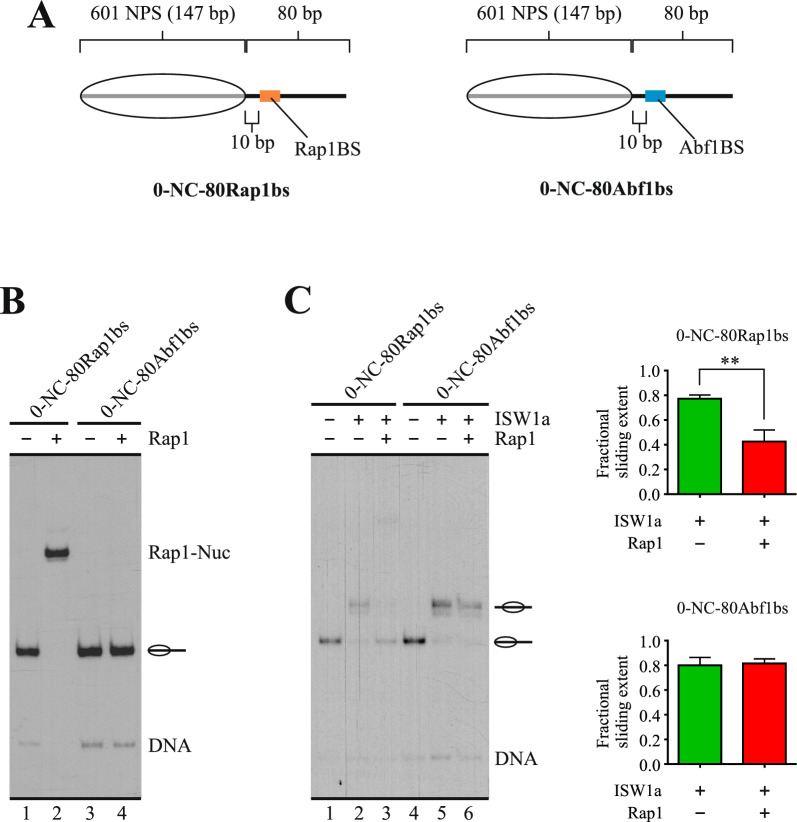
The effect of Rap1 on ISW1a activity requires binding of Rap1 to its cognate sequence. **A** Schematic representation of the nucleosome probes used in the assays. 601 NPS = nucleosome positioning region of the 601 sequence (gray bar; 147 bp). The oval represents the translational position adopted by the nucleosome core upon reconstitution, which covers the 601 region. The term “NC” in probe names stands for “nucleosome core”, while Rap1bs or Abf1bs denote the presence of the target sequence for Rap1 or Abf1, respectively. Probe names indicate length of linker DNA upstream (left) and downstream (right) of the NC. **B** EMSA testing Rap1 binding to the probes shown in “A”. The image corresponds to electrophoresis in a non-denaturing polyacrylamide gel and is representative of three independent assays. The probe used in each reaction and presence of Rap1 (1.4 nM) are depicted at the top of the gel picture; migration of nucleosome probes is indicated schematically at the right of the gel picture, as well as migration of free DNA probes (DNA), and nucleosome probe bound by Rap1 (Rap1-Nuc). **C** Nucleosome remodeling assay visualized by electrophoresis in a non-denaturing polyacrylamide gel, testing the effect of Rap1 on the sliding activity of ISW1a. The probe used in each reaction is depicted at the top of the gel picture, as well as absence or presence of ISW1a (1.5 nM) and Rap1 (1.4 nM). The image is representative of three independent assays, performed under the same conditions. Migrations of alternative forms of the nucleosome probe, which correspond to different translational positions of the histone octamer, are indicated schematically at the right of the picture, as well as migration of free DNA probe (DNA). The graphs at the right correspond to quantification of fractional sliding extent and statistical analysis for each probe. Bars in the graphs display the average of three independent assays for each condition analyzed (n = 3). Error bars represent one standard deviation. Asterisks denote statistically significant differences (**p < 0.01), as deducted from a two-tailed unpaired *t*-test

The high affinity and low dissociation rate of Rap1, relative to Reb1, Abf1 and Cbf1, suggest that these properties underlie the differential effect that Rap1 exerts on ISW1a’s activity. Alternatively, Rap1 could hinder ISW1a’s activity by mechanisms that do not require binding of this factor to its cognate sequence. To test this possibility, we performed an assay testing whether Rap1 is able to hinder ISW1a sliding activity when there is no binding of this GRF to the nucleosomal probe. To do this, we performed a nucleosome remodeling assay to test the effect of Rap1 on the sliding activity of ISW1a using two different nucleosomal probes, one harboring the target sequence of Rap1 and the other harboring the Abf1 target sequence (Fig. 4A). Our results show that Rap1 does not bind to the probe that harbor the Abf1 target sequence (Fig. 4B, compare lane 2 to lane 4) and is not able to hinder ISW1a’s sliding activity on this probe (Fig. 4C, compare lanes 2 and 3 to 5 and 6).

**Fig. 5:**
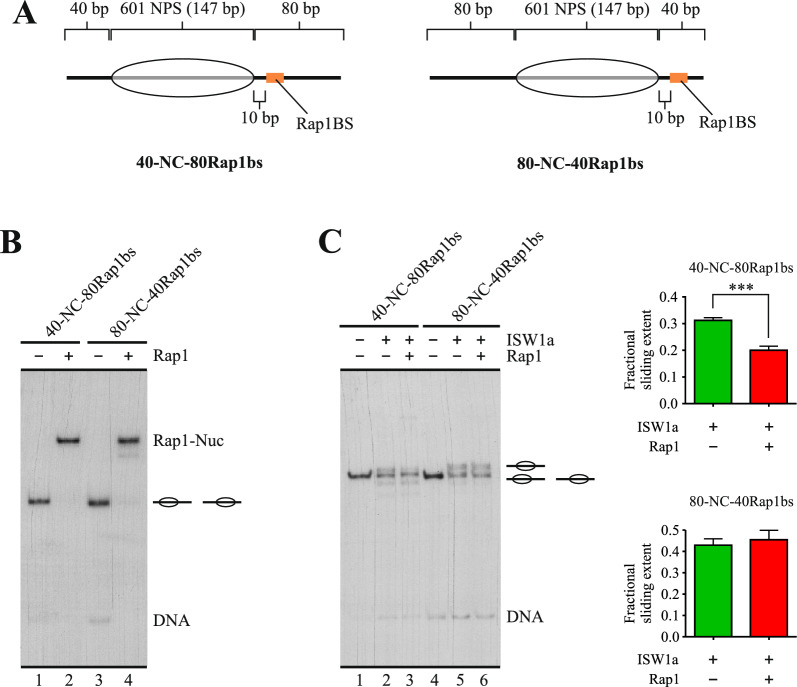
Hindering of ISW1a’s sliding activity by Rap1 requires its binding to linker that becomes entry DNA in the sliding process. **A** Schematic representation of the nucleosome probes used in the assays. 601 NPS = nucleosome positioning region of the 601 sequence (gray bar; 147 bp). The oval represents the translational position adopted by the nucleosome core upon reconstitution, which covers the 601 region. The term “NC” in probe names stands for “nucleosome core”, while Rap1bs denote the presence of the target sequence for Rap1. Probe names indicate length of linker DNA upstream (left) and downstream (right) of the NC. **B** EMSA testing Rap1 binding to the probes shown in “A”. The image corresponds to electrophoresis in a non-denaturing polyacrylamide gel and is representative of three independent assays. The probe used in each reaction and presence of Rap1 (1.4 nM) are depicted at the top of the gel picture; migration of nucleosome probes is indicated schematically at the right of the gel picture, as well as migration of free DNA probes (DNA), and nucleosome probe bound by Rap1 (Rap1-Nuc). **C** Nucleosome remodeling assay visualized by electrophoresis in a non-denaturing polyacrylamide gel, testing the effect of Rap1 on the sliding activity of ISW1a. The probe used in each reaction is depicted at the top of the gel picture, as well as absence or presence of ISW1a (1.5 nM) and Rap1 (1.4 nM). The image is representative of three independent assays, performed under the same conditions. Migrations of alternative forms of the nucleosome probe, which correspond to different translational positions of the histone octamer, are indicated schematically at the right of the picture, as well as migration of free DNA probe (DNA). The graphs at the right correspond to quantification of fractional sliding extent and statistical analysis for each probe. Bars in the graphs display the average of three independent assays for each condition analyzed (n = 3). Error bars represent one standard deviation. Asterisks denote statistically significant differences (***p < 0.001), as deducted from a two-tailed unpaired *t*-test

This result demonstrated that Rap1 requires binding to its target sequence to hinder the sliding activity of ISW1a, which is consistent with a barrier function against nucleosome mobilization in *cis* at NDRs [4, 13]. As this function implies binding of the barrier factor on the DNA that becomes entry DNA in the nucleosome sliding process, we tested whether Rap1’s ability to hinder ISW1a’s activity requires binding of this factor specifically on the entry linker DNA. To do this, we performed remodeling assays comparing two probes. Both probes harbor the Rap1 binding site downstream the nucleosome core and both harbor a long linker (80 bp) on one side and a short linker (40 bp) in the other side. The Rap1 binding site is located in the long linker in one of the probes, while this binding site is located in the short linker in the other (Fig. 5A). The differences in linker length did not affect Rap1 binding (Fig. 5B). As mentioned above, ISW1a preferentially binds to—and mobilizes the histone octamer towards—the longer linker in mononucleosomes. This mobilization continues until the histone octamer reaches a central position relative to the DNA segment, which is reflected by a slower migration of the nucleosome in native gel electrophoresis [19]. Thus, in the probe harboring the Rap1 binding site in the longer linker, ISW1a mobilizes the histone octamer towards the linker DNA where Rap1 is bound, while in the probe that harbors the Rap1 binding site in the shorter linker, ISW1a mobilizes the histone octamer away from the linker DNA where this factor is bound. The result of this analysis confirmed that hindering of ISW1a’s sliding activity by Rap1 requires binding of this factor specifically to the entry linker DNA (Fig. 5C, compare lanes 2 and 3 to 5 and 6).

### Rap1 hinders histone octamer transfer towards DNA where it is bound

**Fig. 6:**
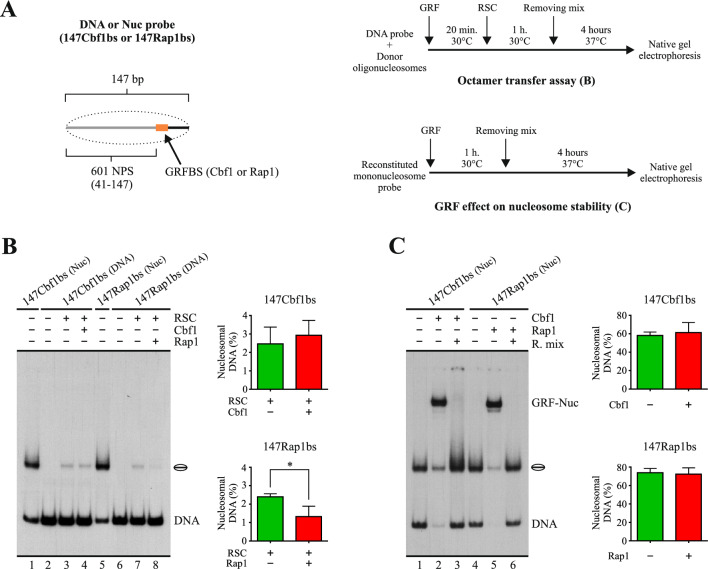
Nucleosome assembly by octamer transfer is hindered by Rap1. **A** Left panel: schematic representation of the nucleosome probes used in the assays. 601 NPS = 107 bp segment of the nucleosome positioning region of the 601 sequence. The dashed oval represents nucleosome core formation upon octamer transfer. Probe names indicate their total length and presence of a defined GRF binding site (GRFbs). Right upper panel: outline of the steps involved in the octamer transfer assay shown in **B**. Right lower panel: outline of the steps involved in the assay testing the effect of GRFs on nucleosome stability shown in **C**. **B** Octamer transfer assay visualized by electrophoresis in a non-denaturing polyacrylamide gel. The probe used in each reaction is depicted at the top of the gel picture, as well as absence or presence of RSC (4 nM), Cbf1 (15 nM) and Rap1 (4 nM). The image is representative of three independent assays, performed under the same conditions. Migration of the nucleosome core is indicated schematically at the right of the picture, as well as migration of free DNA probe (DNA). The graphs at the right correspond to quantification of the extent of nucleosome formation (percentage of nucleosomal DNA) and statistical analysis for each probe. Bars in the graphs display the average of three independent assays for each condition analyzed (n = 3). Error bars represent one standard deviation. Asterisks denote statistically significant differences (*p < 0.05), as deducted from a two-tailed unpaired *t*-test. **C** Effect of Rap1 (or Cbf1) binding on nucleosome stability, as measured by variations in percentage of nucleosomal DNA. The graphs at the right correspond to quantification of percentage of probe at the form of nucleosome. Bars in the graphs display the average of three independent assays for each condition analyzed (n = 3). Error bars represent one standard deviation. See legend of **B** for a general description of the gel image. GRF-Nuc: migration of nucleosome probe bound by Rap1 or by Cbf1

Our results showed that, among the GRFs tested, only Rap1 efficiently hinders nucleosome sliding activity, with this property relying on its binding to the linker that becomes entry DNA during the sliding process. In addition, the ability to contribute to the establishment and maintenance of NFRs might also rely on protecting these regions from histone deposition. In this respect, it has been shown that the action of Rap1 as a transcriptional activator proceeds without triggering histone exchange in the region where the factor is bound and that this property would rely on the activation domain of this factor rather than on its DBD [20]. However, the high affinity of this GRF for its target sequence and its low dissociation rate suggest that these properties, which reside on its DBD, might make Rap1 an efficient competitor against the arrival of histones in the regions where this factor is bound, as previously proposed for NDFs [12]. This effect could also result in a reduced histone exchange rate in these regions by limiting histone deposition. Considering this, we aimed to test the ability of Rap1 to hinder the association of histones to a 147 bp DNA probe when the factor is already bound to this probe. To do this, we performed an octamer transfer assay, where octamer transfer was mediated by the RSC complex [21]. Two different 147 bp probes were compared, one harboring the Rap1 binding site and the Cbf1 binding site the other. The midpoint of the binding site is 32–33 bp from one of the ends of the probes. Thus, the length of the probes forces the binding site to become part of the nucleosome core to be formed (Fig. 6A). The extent of octamer transfer was determined by quantifying the percentage of the probe at the form of nucleosomal DNA. As observed in Fig. 6B, for the probe harboring the Cbf1 binding site, the extent of octamer transfer to this probe was the same in the absence and in the presence of Cbf1 (Fig. 6B, compare lane 3 to 4). On the other hand, for the probe harboring the Rap1 binding site, the presence of Rap1 results in a reduced extent of octamer transfer towards the probe (Fig. 6B, compare lane 7 to 8). Under our assay conditions, both GRFs have the ability to bind to their corresponding target sequence when embedded in a nucleosome core. However, their binding does not result in nucleosome disassembly (Fig. 6C, compare lane 1 to 3 and lane 4 to 6), demonstrating that the reduced extent of nucleosome formation in the presence of Rap1 is not generated by Rap1-mediated disassembly of nucleosomes being formed on the probe during the octamer transfer reaction.

### Binding strength of Rap1 inversely correlates with nucleosome occupancy and histone deposition in vivo

**Fig. 7:**
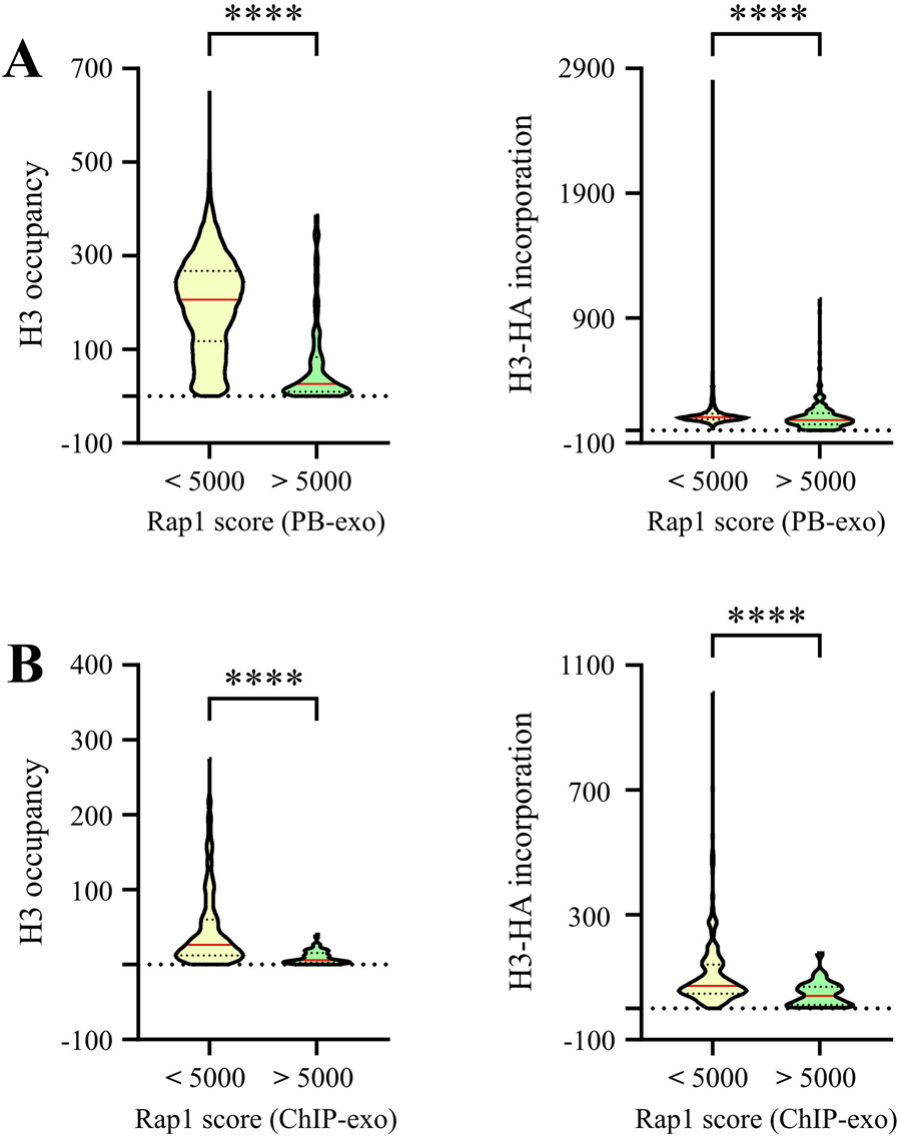
Binding strength of Rap1 inversely correlates with nucleosome occupancy and histone deposition in vivo. **A** Violin plots comparing the distribution of nucleosome occupancy (left panel) and histone deposition (right panel) levels for *loci* displaying low and high binding affinity of Rap1. Nucleosome occupancy and histone deposition levels were determined from genome-wide ChIP-seq data obtained in the study performed by Kassem et al. [20]. An analysis of protein binding to purified genomic DNA coupled to deep sequencing (PB-exo), performed by Rossi et al. [16], was used to define affinities of Rap1 to its target sequences genome-wide. The scores of Rap1 binding strength were divided into low (< 5000) and high (> 5000) affinity clusters. Asterisks denote statistically significant differences (****p < 0.0001), as deducted from the Mann–Whitney *U* test. **B** Violin plots comparing the distribution of nucleosome occupancy (left panel) and histone deposition (right panel) levels for *loci* displaying in vivo low and high occupancy of Rap1. Nucleosome occupancy and histone deposition levels were determined from genome-wide data obtained in the study performed by Kassem et al. [20]. The in vivo genome-wide occupancy levels of Rap1 were obtained from a ChIP-exo analysis performed by Rossi et al. [16]. The Rap1 occupancy scores were divided into low (< 5000) and high (> 5000) affinity clusters. Asterisks denote statistically significant differences (****p < 0.0001), as deducted from the Mann–Whitney *U* test

Our biochemical analyses point to the high affinity of Rap1 to its cognate sequence as one of the key properties underlying its blocking effect on the sliding activity of ISW1a and on octamer transfer. Considering this, we wanted to analyze whether there is a connection between affinity of Rap1 to its target sequences in the *S. cerevisiae* genome and in vivo patterns of nucleosome occupancy and histone deposition. To do this, we performed a bioinformatics analysis comparing publicly available data generated by two different studies. One of them corresponds to in vitro and in vivo genome-wide binding profiles of Rap1 obtained by Rossi et al. (PB-exo and ChIP-exo, respectively; [16]). The other study, performed by Kassem et al., corresponds to genome-wide analyses of nucleosome occupancy and, additionally, replication- and transcription-independent histone deposition [20]. This study determined nucleosome occupancy by ChiP-seq directed to histone H3 and histone deposition by ChIP-seq directed to H3-HA after induction of this epitope-tagged protein. We first compared binding strength of Rap1 to nucleosome occupancy and histone deposition. Binding strength reflecting exclusively affinity of a TF for its target sequences is given by the PB-exo analysis performed by Rossi and co-workers. Comparison of low affinity to high affinity sites shows a differential distribution of both nucleosome occupancy and histone deposition, with high levels of nucleosome occupancy and histone deposition correlated with low affinity sites (Fig. 7A; Additional file 1: Fig. S3A). Additionally, in vivo occupancy of Rap1 shows a similar correlation, with low Rap1 occupancy levels correlated with high levels of nucleosome occupancy and histone deposition (Fig. 7B; Additional file 1: Fig. S3B). Taken together, these genome-wide patterns and the results of our in vitro analyses point to the affinity of Rap1 for its target sequence, higher than that of the other GRFs for their binding sites, as a key feature underlying its capacity to block ISW1a’s sliding activity and histone deposition.

## Discussion

In this work, we performed a comparative analysis of the main GRFs of *S. cerevisiae* with focus on their effect on the nucleosome remodeling activity of the ISW1a complex. Among the GRFs tested in our assays, Rap1 stands as the only factor capable of hindering the sliding activity of this complex. In addition, Rap1 blocks nucleosome formation in an in vitro assay that mimics histone deposition. Concurrently, Rap1 displayed the highest affinity for and longest dwell time from its cognate sequence, compared to the other GRFs tested. Consistently, our bioinformatics analyses show that Rap1 affinity to its target sites in the genome inversely correlates with both nucleosome occupancy and histone deposition at these *loci*.

The results of our current study also show that Rap1 requires the presence of its target sequence to hinder the sliding activity of ISW1a, suggesting that the effect of Rap1 on ISW1a is not mediated by direct protein–protein interactions between this GRF and the complex. Even more, the occurrence of the hindering effect on ISW1a’s sliding activity by Rap1 depends on the location of its target sequence relative to the nucleosome core, with this effect only occurring if this sequence is located in the linker that becomes entry DNA upon ISW1a-mediated remodeling. This fact also makes unlikely a mechanisms based on emergence of protein–protein interactions between Rap1 and ISW1a upon Rap1 binding to its cognate sequence, rather suggesting that the effect of Rap1 is mediated by interfering the interaction of the HSS of ISW1 and/or interaction of Ioc3 with the entry DNA of the sliding process [19, 22].

In addition to the ability of Rap1 to hinder nucleosome sliding activity, the results of our bioinformatics analyses and octamer transfer assays suggest that this factor also contributes to reduction of nucleosome occupancy by hindering histone deposition. In this regard, we have recently found new mechanisms by which poly(dA:dT) tracts enhance nucleosome eviction mediated by the RSC complex [10]. Interestingly, a study performed by Kubik et al. found that binding sites for the Rsc3/Rsc30 subunits of RSC and poly(dA:dT) tracts are commonly downstream Rap1 sites at gene regulatory regions [23]. Taken together, these findings suggest that, at gene regulatory regions harboring this arrangement of regulatory sequences, Rap1 would play the role of hindering histone deposition upon nucleosome eviction mediated by RSC. In addition, Rap1 might assist RSC in the process of chromatin opening [14].

Our results point to the high affinity of Rap1 to its target sequence and its long dwell time, relative to the other GRFs tested, as the features beneath its ability to hinder ISW1a’s sliding activity and histone deposition. As mentioned above, both effects would be contributing to Rap1-mediated reduction of nucleosome occupancy at gene regulatory regions, a role found for Rap1 in early studies addressing its involvement in chromatin dynamics [24]. More recently, it has been shown that enrichment of Rap1 at low nucleosome occupancy *loci* is higher than that observed for Reb1 and Abf1 [25]. Dependence of Rap1 on its high affinity to its binding site, relative to the other GRFs, to hinder histone deposition and nucleosome sliding is supported by our bioinformatics analyses, since they show that nucleosome occupancy and histone deposition are higher at *loci* harboring low affinity Rap1 sites. In this regard, high, medium and low affinity sites have been previously described for Rap1 in the genome of *S. cerevisiae* [26], with long dwell times observed for this GRF only at high and medium affinity sites [14]. Importantly, these evidences suggest that the other GRFs tested in our study might also display properties such as hindering of ISW1a-mediated nucleosome sliding if there were binding sites for these GRFs displaying higher affinity levels than those present in the probes tested. In this regard, it has been demonstrated that variations in sequences flanking target sites of GRFs such as Reb1, deeply alter the affinity of the GRF for these seemingly equal target sites, with flanking sequences granting the highest affinity at NDRs [16].

## Conclusions

Taken together, the findings of our study point to DNA binding affinity and residence time as the key properties of GRFs for hindering nucleosome sliding and assembly, and location of the GRF target sequence at linker that becomes entry DNA of the nucleosome remodeling process as a requirement for hindering the sliding activity of the ISW1a complex. Thus, these features stand out among those that rule the effects that a GRF has on nucleosome dynamics at a particular gene regulatory region.

## Methods

### Recombinant proteins and protein complexes

For each GRF studied in this work, the coding sequence was obtained by PCR on genomic DNA, generating appropriate restriction sites flanking the CDS for cloning into pQE-80L. The PCR product was first cloned into pCR-Blunt II TOPO vector. From this vector, the corresponding flanking restriction sites were used to subclone the CDS into pQE-80L. The sequence of the recombinant vectors generated was confirmed by Sanger sequencing. The recombinant proteins were expressed in *E. coli* BL-21 and purified as N-terminal His-fusion proteins using Ni–NTA agarose resin (cat. 30210, Qiagen), according to the manufacturer’s instructions. The eluted fractions were supplemented with glycerol (15% final concentration) and stored at − 80 °C until their use. Each GRF was eluted under a specific imidazole concentration. This concentration was brought to the same level and then the proteins were diluted in the same extent using TF dilution buffer [10 mM HEPES–KOH (pH 7.4), 100 mM KCl, 1 mM DTT, 15% Glycerol, 10 µM ZnCl_2_, 100 µg/mL BSA, 0.5 mM PMSF, 5 μg/mL leupeptin, 1 μg/mL pepstatin A] to generate the working stock of each GRF. An SDS-PAGE analysis of the purified His-tagged proteins is in Additional file 1: Fig. S1.

The ISW1a and RSC complexes were obtained by tandem affinity purification from Ioc3-TAP and Rsc2-TAP *S. cerevisiae* strains, respectively (Open Biosystems), as previously described [27]. The purified complexes were analyzed as previously reported by us [28]. For each complex, an aliquot of a purification was extensively concentrated (Microcon Ultracel YM-10, Amicon-Millipore), quantified by SDS–PAGE followed by Coomassie staining and then used as standard for Western blot quantification of the purified complex in that and further purifications.

### DNA probes and nucleosome reconstitution

DNA probes of different lengths and harboring the 601 nucleosome positioning sequence located at different positions were generated by PCR using distinct plasmids as templates. The 147 bp positioning region of the 601 sequence was defined as previously described [29]. In the case of probes bearing a binding site for a GRF, for each one the corresponding plasmid was generated by introducing a cassette harboring the binding site. In all these vectors the 601 sequence is separated from the GRF binding site by 10 bp. The plasmids and primer sets used are depicted in Additional file 1: Table S1. Before PCR amplification, one of the primers used in each reaction was labeled on its 5’ end using [γ-^32^P]-ATP (Perkin-Elmer NEG035C or ARC ARP0102B). Nucleosome reconstitution was carried out by the octamer transfer method, as previously described [10]. Oligonucleosomes used as histone donors for reconstitution were obtained from HeLa cells as described elsewhere [30]. All the reconstitution reactions were carried out using 0.5 pmol of probe and 1.5 µg of oligonucleosomes. Once reconstituted, the nucleosome probe (and mock-reconstituted probe) concentration is 4 fmol/μL and the non-labeled oligonucleosomes concentration is 12 ng/μL (in terms of DNA content).

### Binding assays

In each binding reaction, a mix containing 7.9 μL of Remodeling buffer, 0.6 μL of ddH_2_O, 0.5 μL of GRF or TF buffer, 0.5 μL TE buffer, 3 μL of CRC buffer and 2.5 μL of probe was incubated for 30 min at 30 °C (see the composition of each solution used in the mixes in Additional file 1). The final concentration of the different components of this mixture was: 10.7 mM HEPES–KOH pH 7.9, 4 mM Tris–Cl (pH 7.4–8.0), 80.9 mM NaCl, 19.1 mM KCl, 5.4 mM MgCl_2_, 0.2 mM Mg(CH_3_COO)_2_, 0.05% NP-40, 10% glycerol, 0.2 μM ZnCl_2_, 0.2 mM EDTA (pH 8.0), 0.4 mM EGTA, 100 μg/mL BSA, 1.4 mM imidazole, 2 mM DTT, and 0.5 mM PMSF. The samples were then subjected to electrophoresis in non-denaturing polyacrylamide gel (200 V, 0.3 × TBE, 5% acrylamide, 40:1 AA:Bis proportion) in cold room. Afterwards, the gel was dried and autoradiographed on film or scanned using a phosphor screen and Molecular Imager FX (BioRad, Hercules, CA, USA). Densitometric analyses were performed using Quantity One software, v4.1.1 (for phosphor imager files) or UN-SCAN-IT software, v6.1 (films). The extent of binding was calculated as the ratio of bound nucleosome band signal over the combined signal of bound and unbound nucleosome bands in the lane.

For the analysis of dissociation kinetics, the binding reaction proceeded as described for the binding analyses, but scaled 6 times in volume. After binding incubation, a 15 μL aliquot was taken and 13 μL subjected to electrophoresis as described above. To the rest of the sample, 7.5 μL of a mix containing 2.5 μg of non-labeled DNA (ladder DNA, NEB N3231S, further purified by organic extraction) and a non-labeled double-stranded oligonucleotide harboring the GRF’s target sequence (100× final concentration in the reaction mix, relative to GRF concentration) was added, and the incubation at 30 °C was continued for 60 min. During this incubation time, 13 μL aliquots were taken at defined time points (10, 20, 30 and 60 min) and immediately loaded in the gel. Gel drying and subsequent analyses proceeded as described above.

### Nucleosome sliding assays

A mix containing 7.9 μL of Remodeling buffer, 0.6 μL of ATP (Roche, 11140965001), 0.5 μL of GRF or TF buffer, 0.5 μL TE buffer, and 2.5 μL of probe was incubated for 20 min at 30 °C (see the composition of each solution used in the mixes in Additional file 1). Then, purified ISW1a complex (brought to 3 μL using CRC buffer) or CRC buffer (3 μL) was added, incubating for additional 45 min at 30 °C. After this incubation, 1.5 μL of a mix containing 500 ng of non-labeled DNA (ladder DNA, NEB N3231S, further purified by organic extraction) and a non-labeled double-stranded oligonucleotide harboring the corresponding GRF’s target sequence (100× final concentration in the reaction mix, relative to GRF concentration) was added (removing mix), incubating for 2–4 h at 37 °C. The samples were then subjected to gel electrophoresis. This step and further steps proceeded as described for binding assays. The extent of nucleosome sliding was calculated as the ratio of the signal of slid mononucleosome band over the signal of all mononucleosome bands in the lane.

### Octamer transfer assays

A mix containing 7.9 μL of Remodeling buffer-OT, 0.6 μL of ATP (Roche, 11140965001), 0.5 μL of GRF or TF buffer-OT, 0.5 μL of oligonucleosomes (in FCR buffer), and 2.5 μL of probe was incubated for 20 min at 30 °C (see the composition of each solution used in the mixes in Additional file 1). Then, purified RSC complex (brought to 3 μL using CRC buffer) or CRC buffer (3 μL) was added, incubating for 1 h at 30 °C. The final concentration of the different components of this mixture was: 10.7 mM HEPES–KOH pH 7.9, 4 mM Tris–Cl (pH 7.4–8.0), 51.8 mM NaCl, 48.2 mM KCl, 5.4 mM MgCl_2_, 0.2 mM Mg(CH_3_COO)_2_, 0.05% NP-40, 10% glycerol, 0.26 μM ZnCl_2_, 0.2 mM EDTA (pH 8.0), 0.4 mM EGTA, 100 μg/mL BSA, 0.64 mM imidazole, 2 mM DTT, and 0.5 mM PMSF. After this incubation, 1.5 μL of the Removing mix described above for the sliding assays was added, followed by a 4 h incubation at 37 °C. The samples were then subjected to gel electrophoresis. This step and further steps proceeded as described for binding assays. The extent of nucleosome assembly was calculated as the ratio of nucleosome band signal over the combined signal of nucleosome and naked DNA bands in the lane.

### Bioinformatics analyses

The bigwig files corresponding to H3-HA incorporation [H3-HA_TBP-AA_T30 (rep2)] and H3 occupancy [H3_TBP-AA_T30 (rep2)] were obtained from GSE143305 [20]. For both files, the genome was partitioned into 50 bp bins using bigwigAverage from deeptools, to obtain the incorporation or occupancy in these bins. On the other hand, gff files corresponding to Filtered_PeakPairs for ChIP-exo and PB-exo of Rap1 were obtained from GSE93662 [16]. Files were sorted, and then the intersections of Rap1 peaks with H3-HA incorporation or H3 occupancy were performed using bedtools Intersect option -wo. Subsequently, the intersections were analyzed in GraphPad Prism 9 to generate correlation and violin plots. Presence or absence of statistically significant differences was determined using the Mann–Whitney U test.

### Supplementary Information

**Additional file 1.** Detailed information regarding methods. **Figure S1.** SDS-PAGE analysis of purified His-tagged transcription factors. **Figure S2.** Standardization of Rap1 removal for nucleosome remodeling assays. **Figure S3.** High nucleosome occupancy and histone deposition levels are mainly present at loci displaying low affinity or low occupancy levels of Rap1. **Table S1.** Sequence information of template plasmid and primers used for generation of each probe. **Table S2.** Yeast strains used for purification of ATP-dependent chromatin remodeling complexes.

## Data Availability

The data presented in this study are available on request from the corresponding author.
