# Supplementary material for: General regulatory factors exert differential effects on nucleosome sliding activity of the ISW1a complex
Source: Biol Res. 2024 May 4;57:22. doi: 10.1186/s40659-024-00500-6 (PMC11069190; doi:10.1186/s40659-024-00500-6)
Supplement: Supplementary file 1 — Additional file 1. Detailed information regarding methods. Figure S1. SDS-PAGE analysis of purified His-tagged transcription factors. Figure S2. Standardization of Rap1 removal for nucleosome remodeling assays. Figure S3. High nucleosome occupancy and histone deposition levels are mainly present at loci displaying low affinity or low occupancy levels of Rap1. Table S1. Sequence information of template plasmid and primers used for generation of each probe. Table S2. Yeast strains used for purification of ATP-dependent chromatin remodeling complexes. [file 40659_2024_500_MOESM1_ESM.pdf]

Additional file 1

Methods

Composition of buffers mixed in binding and nucleosome sliding assays

The final concentration of each component in a reaction mix is given in the Materials and Methods section of the main text.

Remodeling buffer (7.9 µL): 20 mM HEPES-KOH (pH 7.9), 33.06 mM KCl, 0.57 mM PMSF, 1.97 mM DTT, 0.04 % NP-40, 11.07 % Glycerol, 10.25 mM $MgCl_2$, 155.05 µg/mL BSA.

His-protein or TF buffer (0.5 µL): 5 mM HEPES-KOH (pH 7.4), 127.5 mM NaCl, 50 mM KCl, 21.25 mM NaH_2PO_4, 15 % Glycerol, 5 µM $ZnCl_2$, 35 mM Imidazole, 50 µg/mL BSA, 0.93 mM DTT, 0.47 mM PMSF, 5µg/mL leupeptin, 1µg/mL pepstatin A.

Chromatin remodeling complex or CRC buffer (3 µL): 10 mM Tris-Cl (pH 8.0), 300 mM NaCl, 1 mM $Mg(CH_3COO)_2$, 1 mM Imidazole, 2 mM EGTA, 0.1 % NP-40, 0.5 mM PMSF, 0.5 mM DTT, 10 % Glycerol, 5µg/mL leupeptin, 1µg/mL pepstatin A.

TE buffer (0.5 µL): 10 mM Tris-Cl (pH 8.0), 1 mM EDTA.

Deionized water or ATP (0.6 µL): 50 mM ATP.

Probe (2.5 µL): 10 mM Tris-Cl (pH 7.4), 1 mM EDTA, 0,5 mM PMSF, 5 mM DTT, 0,05% NP-40, 10 % Glycerol, 100 µg/mL BSA, 100 mM NaCl.

Total reaction volume: 15 µL.

Composition of buffers mixed in octamer transfer (OT) assays

The final concentration of each component in a reaction mix is given in the Materials and Methods section of the main text.

Remodeling buffer-OT (7.9 µL): 20 mM HEPES-KOH (pH 7.9), 86.54 mM KCl, 0.54 mM PMSF, 1.65 mM DTT, 0.04 % NP-40, 10.44 % Glycerol, 10.25 mM $MgCl_2$, 146.91 µg/mL BSA.

His-protein or TF buffer-OT (0.5 µL): 7.9 mM HEPES-KOH (pH 7.4), 53.9 mM NaCl, 78.9 mM KCl, 9 mM NaH_2PO_4, 15 % Glycerol, 7.9 µM $ZnCl_2$, 13.1 mM Imidazole, 78.9 µg/mL BSA, 1 mM DTT, 0.5 mM PMSF, 5µg/mL leupeptin, 1µg/mL pepstatin A.

Chromatin remodeling complex or CRC buffer (3 µL): 10 mM Tris-Cl (pH 8.0), 150 mM NaCl, 1 mM $Mg(CH_3COO)_2$, 1 mM Imidazole, 2 mM EGTA, 0.1 % NP-40, 0.5 mM PMSF, 0.5 mM DTT, 10 % Glycerol, 5µg/mL leupeptin, 1µg/mL pepstatin A.

Oligonucleosomes or FCR (Final Conditions of Reconstitution) buffer (0.5 µL): 10 mM Tris-Cl (pH 7.4), 1 mM EDTA, 0,5 mM PMSF, 5 mM DTT, 0,05% NP-40, 10 % Glycerol, 100 µg/mL BSA, 100 mM NaCl.

Deionized water or ATP (0.6 µL): 50 mM ATP.

Probe (2.5 µL): 10 mM Tris-Cl (pH 7.4), 1 mM EDTA, 0,5 mM PMSF, 5 mM DTT, 0,05% NP-40, 10 % Glycerol, 100 µg/mL BSA, 100 mM NaCl.

Total reaction volume: 15 µL.

Figure legends

Figure S1. SDS-PAGE analysis of purified His-tagged transcription factors. The image shows a Coomassie blue-stained gel (12%) of His-tagged proteins expressed in and purified from *E. coli* BL-21. The identity of each purified protein is depicted at the top of the gel picture. MW: molecular weight marker (Unstained Protein Standard, Broad Range, New England Biolabs P7717S). BSA: bovine serum albumin (BioRad 5000206), used for densitometric quantification of the recombinant proteins.

Figure S2. Standardization of Rap1 removal for nucleosome remodeling assays (A) Schematic representation of the nucleosome probe used in the standardization assays. 601 NPS = nucleosome positioning region of the 601 sequence (gray bar; 147 bp). The oval represents the translational position adopted by the nucleosome core upon reconstitution, which covers the 601 region. The term "NC" in probe names stands for "nucleosome core". The probe name indicates length of linker DNA upstream (left) and downstream (right) of the NC and presence of the Rap1 binding site (Rap1bs). **(B)** Outline of the steps involved in the assays. The Removing mix consist in a mix that contains 500 ng of an unrelated non-labeled DNA and a non-labeled double-stranded oligonucleotide harboring the Rap1's target sequence, added in an excess of 100x, 200x or 300x relative to Rap1 concentration (100x in figure C). **(C)** EMSA testing the effect of temperature on the efficiency of Rap1 removal from its target sequence. The image corresponds to electrophoresis in a non-denaturing polyacrylamide gel. Each temperature was tested in duplicate. The conditions for each reaction are depicted at the top of the gel picture, as well as percentages of bound probe; migration of the nucleosome probe is indicated schematically at the right of the gel picture, as well as migrations of free DNA probe (DNA), and nucleosome probe bound by Rap1 (Rap1-Nuc). **(D)** EMSA testing effect of increasing amounts of competitor oligonucleotide on the efficiency of Rap1 removal from its target sequence in the probe. Removal incubations were performed at 37°C in all reactions of this assay. The image corresponds to electrophoresis in a non-denaturing polyacrylamide gel. The conditions for each reaction are depicted at the top of the gel picture, as well as percentages of bound probe; migration of the nucleosome probe is indicated schematically at the right of the gel picture, as well as migrations of free DNA probe (DNA), and nucleosome probe bound by Rap1 (Rap1-Nuc).

Figure S3. High nucleosome occupancy and histone deposition levels are mainly present at *loci* displaying low affinity or low occupancy levels of Rap1. (A) Scatter plots displaying nucleosome occupancy (left panel) and histone deposition (right panel) levels at *loci* bound by Rap1, relative to affinity levels of Rap1 at these *loci*. Nucleosome occupancy and histone deposition levels were determined from genome-wide ChIP-seq data obtained in the study performed by Kassem and co-workers (1). An analysis of protein binding to purified genomic DNA coupled to deep sequencing (PB-exo), performed by Rossi and co-workers (2), was used to define affinities of Rap1 to its target sequences genome-wide. **(B)** Scatter plots displaying nucleosome occupancy (left panel) and histone deposition (right panel) levels at *loci* bound *in vivo* by Rap1, relative to occupancy levels of Rap1 at these *loci*. Nucleosome occupancy and histone deposition levels were determined from genome-wide ChIP-seq data obtained in the study performed by Kassem and co-workers (1). The *in vivo* genome-wide occupancy levels of Rap1 were obtained from a ChIP-exo analysis performed by Rossi and co-workers (2).

Table S1. Sequence information of template plasmid and primers used for generation of each probe.

Probe name	Plasmid name and sequence harboring the region amplified in PCR reaction	Primers
20-NC-60Reb1bs	p601-10-Reb1<br>5'ggtcgctgttcaatacatgcacaggatgtatatatctgacacgtgcctggagactagggagtaatccc<br>cttggcggttaaaacgcgggggacagcgcgtacgtgcgtttaagcggtgctagagctgtctacgaccaat<br>tgagcggcctcggcaccgggattctccagggcggccgctcgggtaaagcatcaccatggtgatgaccgc<br>ggacctgcaggcatgcaagc	Forward:<br>5'GGTCGCTGTTCAATACAT<br>Reverse:<br>5'GCTTGCATGCCTGCAGGT
20-NC-60Abf1bs	p601-10-Abf1<br>5'ggtcgctgttcaatacatgcacaggatgtatatatctgacacgtgcctggagactagggagtaatccc<br>cttggcggttaaaacgcgggggacagcgcgtacgtgcgtttaagcggtgctagagctgtctacgaccaat<br>tgagcggcctcggcaccgggattctccagggcggccgcttatcgtattgcatgatatggtgatgaccgcg<br>gacctgcaggcatgcaagc	Forward:<br>5'GGTCGCTGTTCAATACAT<br>Reverse:<br>5'GCTTGCATGCCTGCAGGT
20-NC-60Cbf1bs	p601-10-Cbf1<br>5'ggtcgctgttcaatacatgcacaggatgtatatatctgacacgtgcctggagactagggagtaatccc<br>cttggcggttaaaacgcgggggacagcgcgtacgtgcgtttaagcggtgctagagctgtctacgaccaat<br>tgagcggcctcggcaccgggattctccagggcggccgctgtcacgtgggctagtaatggtgatgaccgc<br>ggacctgcaggcatgcaagc	Forward:<br>5'GGTCGCTGTTCAATACAT<br>Reverse:<br>5'GCTTGCATGCCTGCAGGT
20-NC-60Rap1bs	p601-10-Rap1<br>5'ggtcgctgttcaatacatgcacaggatgtatatatctgacacgtgcctggagactagggagtaatccc<br>cttggcggttaaaacgcgggggacagcgcgtacgtgcgtttaagcggtgctagagctgtctacgaccaat<br>tgagcggcctcggcaccgggattctccagggcggccgctacacccatacatttccatggtgatgaccgcg<br>gacctgcaggcatgcaagc	Forward:<br>5'GGTCGCTGTTCAATACAT<br>Reverse:<br>5'GCTTGCATGCCTGCAGGT
0-NC-80Rap1bs	p601-10-Rap1<br>5'acaggatgtatatatctgacacgtgcctggagactagggagtaatccccttggcggttaaaacgcggg<br>ggacagcgcgtacgtgcgtttaagcggtgctagagctgtctacgaccaattgagcggcctcggcaccgg<br>gattctccagggcggccgctacacccatacatttccatggtgatgaccgcggacctgcaggcatgcaagc<br>ttgagtattctatagtgtca	Forward:<br>5'ACAGGATGTATATATCTGACAC<br>GTGCCTGG<br>Reverse:<br>5'TGACACTATAGAATACTCAAGC
0-NC-80Abf1bs	p601-10-Abf1<br>5'acaggatgtatatatctgacacgtgcctggagactagggagtaatccccttggcggttaaaacgcggg<br>ggacagcgcgtacgtgcgtttaagcggtgctagagctgtctacgaccaattgagcggcctcggcaccgg<br>gattctccagggcggccgcttatcgtattgcatgatatggtgatgaccgcggacctgcaggcatgcaagc<br>ttgagtattctatagtgtca	Forward:<br>5'ACAGGATGTATATATCTGACAC<br>GTGCCTGG<br>Reverse:<br>5'TGACACTATAGAATACTCAAGC
40-NC-80Rap1bs	p601-10-Rap1<br>5'tatccgactggcaccggcaaggtcgctgttcaatacatgcacaggatgtatatatctgacacgtgcctg<br>gagactagggagtaatccccttggcggttaaaacgcgggggacagcgcgtacgtgcgtttaagcggtgc<br>tagagctgtctacgaccaattgagcggcctcggcaccgggattctccagggcggccgctacacccataca<br>tttccatggtgatgaccgcggacctgcaggcatgcaagcttgagtattctatagtgtca	Forward:<br>5'TATCCGACTGGCACCG<br>Reverse:<br>5'TGACACTATAGAATACTCAAGC
80-NC-40Rap1bs	p601-10-Rap1<br>5'tcggtacccggggatcctctagagtgggagctcggaacactatccgactggcaccggcaaggtcgctg<br>ttcaatacatgcacaggatgtatatatctgacacgtgcctggagactagggagtaatccccttggcggtta<br>aaacgcgggggacagcgcgtacgtgcgtttaagcggtgctagagctgtctacgaccaattgagcggcct<br>cggcaccgggattctccagggcggccgctacacccatacatttccatggtgatgaccgc	Forward:<br>5'TCGGTACCCGGGGAT<br>Reverse:<br>5'GCGGTCATCACCATG
147Cbf1bs	p601-0-Cbf1<br>5'gtaatccccttggcggttaaaacgcgggggacagcgcgtacgtgcgtttaagcggtgctagagctgtc<br>tacgaccaattgagcggcctcggcaccgggattctccaggtcacgtgggctagtagagcatcaccatggt<br>gatgaccgc	Forward:<br>5'GTAATCCCCTTGGCGGTTA<br>Reverse:<br>5'GCGGTCATCACCATG
147Rap1bs	p601-0-Rap1<br>5'gtaatccccttggcggttaaaacgcgggggacagcgcgtacgtgcgtttaagcggtgctagagctgtc<br>tacgaccaattgagcggcctcggcaccgggattctccagacacccatacattttcgagcatcaccatggt<br>gatgaccgc	Forward:<br>5'GTAATCCCCTTGGCGGTTA<br>Reverse:<br>5'GCGGTCATCACCATG

Letters in red correspond to the nucleosome positioning region of Widom's 601 sequence (3).

Sequences highlighted in yellow correspond to the regions bound by the PCR primers.

Underlined sequences correspond to the binding site of Reb1 (4), Abf1 (5), Cbf1 (6) or Rap1 (7).

Table S2. Yeast strains used for purification of ATP-dependent chromatin remodeling complexes.

Complex (tagged subunit)	Strain	Name	Genotype (background)	Source
**RSC (Rsc2)**	YLR357w	Rsc2-TAP	*RSC2-TAP (BY4741)*	Open Biosystems
**ISW1a (Ioc3)**	YFR013w	Ioc3-TAP	*IOC3-TAP (BY4741)*	Open Biosystems

References

1. Kassem, S., Ferrari, P., Hughes, A.L., Soudet, J., Rando, O.J., and Strubin, M. (2020) Histone exchange is associated with activator function at transcribed promoters and with repression at histone *loci*. *Sci Adv*, **6**, eabb0333.
2. Rossi, M.J., Lai, W.K.M., and Pugh, B.F. (2018) Genome-wide determinants of sequence-specific DNA binding of general regulatory factors. *Genome Res*, **28**, 497-508.
3. Li, G. and Widom, J. (2004) Nucleosomes facilitate their own invasion. *Nat Struct Mol Biol*, **11**, 763-769.
4. Qiu, Y., Fakas, S., Han, G.S., Barbosa, A.D., Siniossoglou, S., and Carman, G.M. (2013) Transcription factor Reb1p regulates DGK1-encoded diacylglycerol kinase and lipid metabolism in *Saccharomyces cerevisiae*. *J Biol Chem*, **288**, 29124-33.
5. Beinoraviciūte-Kellner, R., Lipps, G., and Krauss, G. (2005) *In vitro* selection of DNA binding sites for Abf1 protein from *Saccharomyces cerevisiae*. *FEBS letters*, **579**, 4535–4540.
6. Teytelman, L., Osborne Nishimura, E. A., Özaydin, B., Eisen, M. B., and Rine, J. (2012) The enigmatic conservation of a Rap1 binding site in the *Saccharomyces cerevisiae* HMR-E silencer. *G3*, **2**, 1555–1562.
7. Le, D. D., Shimko, T. C., Aditham, A. K., Keys, A. M., Longwell, S. A., Orenstein, Y., and Fordyce, P. M. (2018) Comprehensive, high-resolution binding energy landscapes reveal context dependencies of transcription factor binding. *Proc Natl Acad Sci USA*, **115**, E3702–E3711.

Figure S1

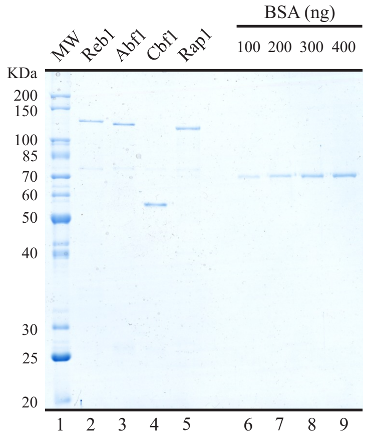
BSA (ng)
MW
Reb1
Abf1
Cbf1
Rap1
100
200
300
400
KDa
200
150
100
85
70
60
50
40
30
25
20
1
2
3
4
5
6
7
8
9

Figure S2

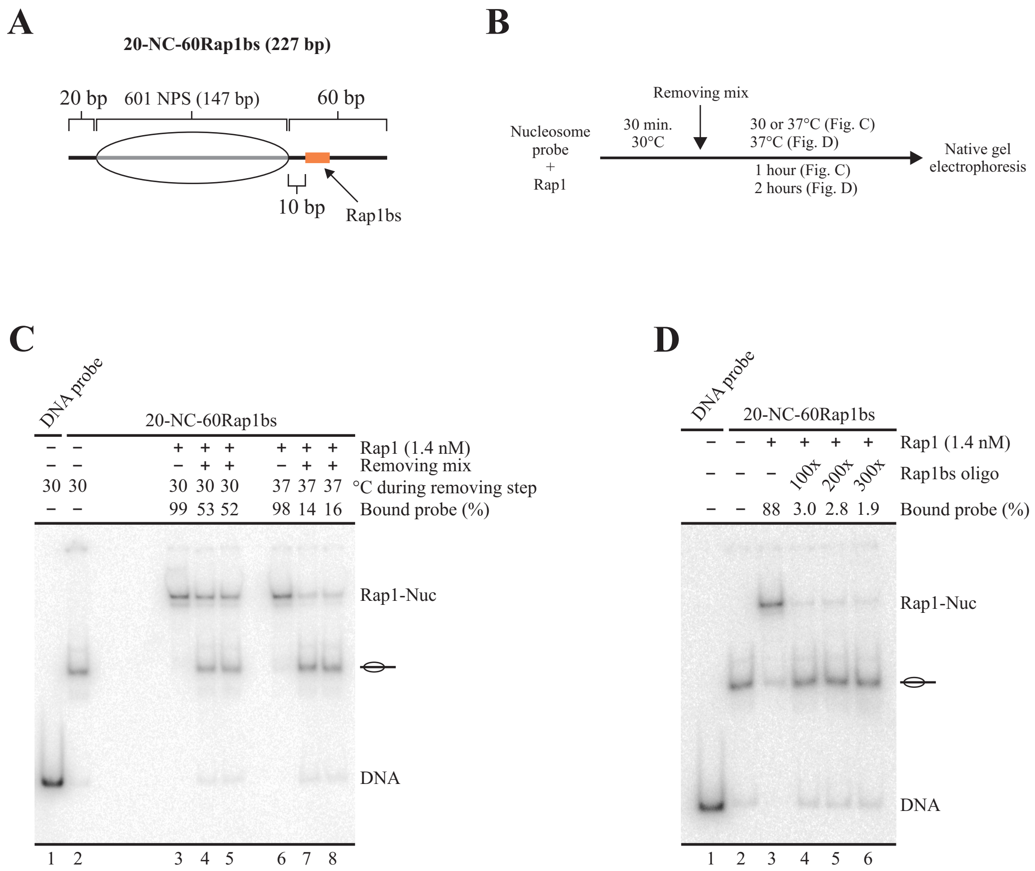
A
20-NC-60Rap1bs (227 bp)
20 bp
601 NPS (147 bp)
60 bp
10 bp
Rap1bs
B
Removing mix
Nucleosome probe + Rap1
30 min. 30°C
30 or 37°C (Fig. C)
37°C (Fig. D)
1 hour (Fig. C)
2 hours (Fig. D)
Native gel electrophoresis
C
DNA probe
20-NC-60Rap1bs
Rap1 (1.4 nM)
Removing mix
°C during removing step
Bound probe (%)
30 30 30 30 30 37 37 37
99 53 52 98 14 16
Rap1-Nuc
DNA
1 2 3 4 5 6 7 8
D
DNA probe
20-NC-60Rap1bs
Rap1 (1.4 nM)
Rap1bs oligo
100x 200x 300x
Bound probe (%)
88 3.0 2.8 1.9
Rap1-Nuc
DNA
1 2 3 4 5 6

Figure S3

A

B

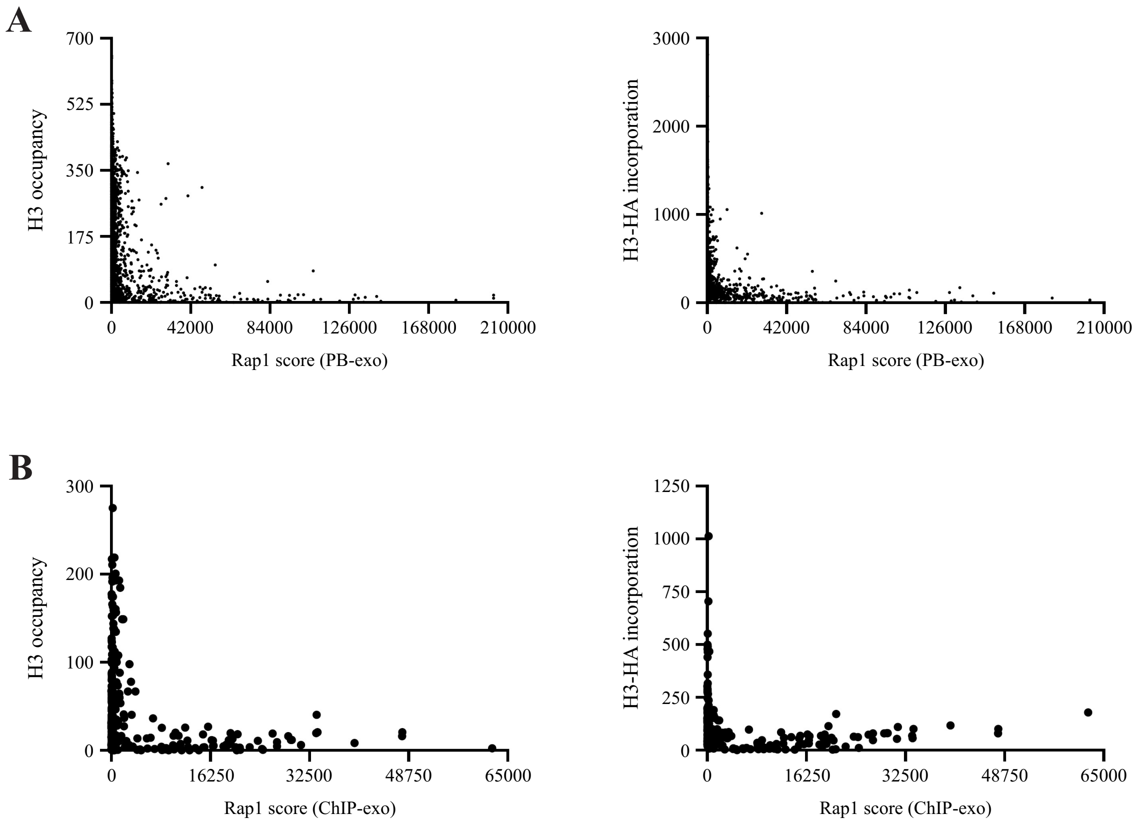
A
H3 occupancy
700
525
350
175
0
0
42000
84000
126000
168000
210000
Rap1 score (PB-exo)
H3-HA incorporation
3000
2000
1000
0
Rap1 score (PB-exo)
B
H3 occupancy
300
200
100
0
16250
32500
48750
65000
Rap1 score (ChIP-exo)
H3-HA incorporation
1250
1000
750
500
250
Rap1 score (ChIP-exo)
